# Phospholipase A2 (PLA_2_) as an Early Indicator of Envenomation in Australian Elapid Snakebites (ASP-27)

**DOI:** 10.3390/biomedicines8110459

**Published:** 2020-10-29

**Authors:** Geoffrey K. Isbister, Nandita Mirajkar, Kellie Fakes, Simon G. A. Brown, Punnam Chander Veerati

**Affiliations:** 1 Clinical Toxicology Research Group, University of Newcastle, Newcastle, NSW 2298, Australia; nanditamirajkar22@gmail.com (N.M.); kellie.fakes@newcastle.edu.au (K.F.); punnam.veerati@newcastle.edu.au (P.C.V.); 2 Aeromedical and Retrieval Medicine, Ambulance Tasmania, Hobart, TAS 7001, Australia; simon.brown@ambulance.tas.gov.au

**Keywords:** snakebite, envenomation, phospholipase, diagnosis, antivenom, venom

## Abstract

Early diagnosis of snake envenomation is essential, especially neurotoxicity and myotoxicity. We investigated the diagnostic value of serum phospholipase (PLA_2_) in Australian snakebites. In total, 115 envenomated and 80 non-envenomated patients were recruited over 2 years, in which an early blood sample was available pre-antivenom. Serum samples were analyzed for secretory PLA_2_ activity using a Cayman sPLA_2_ assay kit (#765001 Cayman Chemical Company, Ann Arbor MI, USA). Venom concentrations were measured for snake identification using venom-specific enzyme immunoassay. The most common snakes were *Pseudonaja* spp. (33), *Notechis scutatus* (24), *Pseudechis porphyriacus* (19) and *Tropidechis carinatus* (17). There was a significant difference in median PLA_2_ activity between non-envenomated (9 nmol/min/mL; IQR: 7–11) and envenomated patients (19 nmol/min/mL; IQR: 10–66, *p* < 0.0001) but *Pseudonaja* spp. were not different to non-envenomated. There was a significant correlation between venom concentrations and PLA_2_ activity (r = 0.71; *p* < 0.0001). PLA_2_ activity was predictive for envenomation; area under the receiver-operating-characteristic curve (AUC-ROC), 0.79 (95% confidence intervals [95%CI]: 0.72–0.85), which improved with brown snakes excluded, AUC-ROC, 0.88 (95%CI: 0.82–0.94). A cut-point of 16 nmol/min/mL gives a sensitivity of 72% and specificity of 100% for Australian snakes, excluding *Pseudonaja*. PLA_2_ activity was a good early predictor of envenomation in most Australian elapid bites. A bedside PLA_2_ activity test has potential utility for early case identification but may not be useful for excluding envenomation.

## 1. Introduction

Snake envenomation is a major health issue and is recognized as a neglected tropical disease, particularly throughout South and South-East Asia, sub-Saharan Africa and Indonesia [1,2]. Antivenom remains the only specific treatment for snake envenomation [3], and there is increasing evidence supporting the greater effectiveness of early antivenom [4,5,6,7]. This is particularly important for preventing neurotoxicity and myotoxicity, which are irreversible effects of snake venom [4,6,7,8]. In Australia, myotoxicity and neurotoxicity can occur from bites by most medically important snakes, including *Notechis* spp. (tiger snakes), *Pseudechis* spp. (black snakes), *Tropidechis carinatus* (Rough-scaled snake), *Oxyuranus scutellatus* (taipans) and *Ancanthophis* spp. (death adders), but not from *Pseudonaja* spp. (brown snakes).

Determining if patients are envenomated within hours of a snakebite is difficult and often relies on the presence of non-specific systemic symptoms, such as headache, nausea, vomiting and abdominal pain [9]. Bedside and laboratory coagulation studies are also often used for early diagnosis in many viper bites and Australian elapid bites, because procoagulant toxins are common [10]. Unfortunately, the commonly used 20-min whole blood clotting test (WBCT20) is not sensitive enough [11,12] and laboratory assays, such as a prothrombin time (PT/international normalized ratio [INR]), are not readily available and can delay patient assessment, even in regions in which these assays are readily available. Neurotoxicity and myotoxicity are difficult to predict early, and readily available biomarkers, such as creatine kinase, lag the tissue damage by many hours and cannot be relied upon [13].

A potential approach for assessing patients for envenomation is to have a laboratory or, ideally, a bedside assay that detects the presence of a common snake toxin in blood. The particular toxin would need to be present in most snake venoms and be easily detectable. Phospholipase A_2_ (PLA_2_) occurs in the venoms of almost all venomous snakes, including major groups of vipers and elapids [14]. The presence of PLA_2_ toxins can be detected by measuring the secretory PLA_2_ activity. A previous study found that PLA_2_ activity was elevated early in viper envenomation compared to non-envenomated snakebite patients [15]. Although the PLA_2_ toxin may not be the medically important toxin in all snake venoms, its presence in serum indicates that venom has reached the central compartment and the patient has systemic envenomation.

A large Australian study recently demonstrated that there continue to be important delays in the administration of antivenom [16], despite evidence that antivenom should be administered as early as possible. The availability of an early diagnostic assay, such as measurement of PLA_2_ activity, could potentially improve outcomes in snakebite. In particular, it could identify patients with bites from snakes known to cause myotoxicity or neurotoxicity so that antivenom can be given within 2 to 3 h.

In this study, we investigate the diagnostic value of measuring PLA_2_ in a cohort of elapid snakebite patients from across Australia. We aimed to determine if early detection of PLA_2_ in patient serum was associated with envenomation, compared to patients without envenomation.

## 2. Experimental Section

We undertook a study of snakebites recruited to the Australian Snakebite Project (ASP) to investigate if the measurement of PLA_2_ activity identified patients with systemic envenomation. ASP is a prospective observational study of suspected and definite snakebites from over 200 Australian hospitals. We have previously published the design, recruitment strategies and data collection for ASP [16]. Approval for the study has been obtained from Human Research and Ethics Committees covering all institutions involved.

We identified snakebite cases from hospitals around Australia via calls to a national free call number, calls to the National Poison Centre Network and calls from local investigators. All patients recruited to ASP had the following data collected: demographics, bite circumstances, clinical effects, laboratory investigations, complications and treatment. We obtained data from datasheets faxed with consent forms to each treating hospital. These were filled out by the clinicians and faxed back to us. Any missing data were obtained from the hospital medical records as required. A trained research assistant entered the data into a relational database (Microsoft Access™), which was reviewed by the chief investigator.

Systemic envenomation in ASP is defined as a patient having one or more of the previously defined Australian clinical envenomation syndromes based on clinical features and laboratory testing (Table 1) [16]. Patients were determined to be non-envenomated if they did not develop any of the clinical envenomation syndromes for at least 12 h post-bite [17]. We identified the snake type using venom-specific enzyme immunoassay performed on blood in patients with systemic envenomation, or from expert identification by a licensed reptile handler or a professional working with snakes at a zoo or museum.

For this study, we included patients recruited from July 2015 to June 2017 with a reported snakebite, in which there was an early blood sample available prior to the administration of antivenom. All cases were then determined to be envenomated (with systemic envenomation) or non-envenomated.

The first serum sample collected for each patient was analyzed for secretory PLA_2_ activity by Cayman’s PLA_2_ assay kit (#765001Cayman Chemical Company, Ann Arbor, MI, USA), according to the manufacturer’s instructions. Serum samples and a premix solution containing assay buffer and an indicator, DTNB [5,5′-dithio-bis-(2-nitrobenzoic acid)], were added to wells of a 96-well plate. A substrate solution containing Dihepanoyl Thio-PC was then added to each well. The assay plate was immediately transferred to a spectrophotometer (SynergyTM HT Multi-Detection Micoplate Reader, BioTek) to read the samples every minute at a 414-nm wavelength for a yellow color change. The absorbance values were then used to calculate the sPLA_2_ activity (µmol/min/mL) in each sample.

Venom concentrations were measured in all envenomated patients with a venom-specific enzyme immunoassay as previously described [18]. Rabbits were used to raise polyclonal IgG antibodies against eight Australasian elapid venoms (*Pseudonaja* spp., *Pseudechis australis*, *P. porphyriacus*, *Notechis scutatus*, *Tropidechis carinatus*, *Oxyuranus scutellatus*, *Acanthophis antarcticus* and *Hoplocephalus stephansii*). Antibodies were then bound to the microplate wells and also conjugated with biotin for detection in a sandwich enzyme immunoassay. The detecting agent was streptavidin-horseradish peroxidase. Each sample was assayed in triplicate (coefficient of variation of <10%), and averaged absorbances were then converted to venom concentrations using a standard curve. The limit of detection of the eight assays ranged from 0.1 to 0.2 ng/mL.

All continuous variables were reported with medians, interquartile ranges (IQR) and ranges. We compared the PLA_2_ activity between envenomated and non-envenomated patients and between different groups of snakes, using the Kruskal-Wallis test. We investigated any association between venom concentration (venom load) and PLA_2_ activity by testing with Pearson correlation analysis. The predictive performance of the PLA_2_ activity in diagnosing systemic envenomation was tested using area under the receiver-operating-characteristic curve (ROC-AUC). We examined the sensitivity, specificity and likelihood ratio of PLA_2_ activity in diagnosing systemic envenomation. Further analysis was undertaken to determine if PLA_2_ activity was correlated with myotoxicity, a toxic effect known to be caused by PLA_2_ toxins. We undertook all analyses and produced graphs using GraphPad Prism version 8.2 for Windows (GraphPad Software, San Diego, CA, USA, www.graphpad.com).

## 3. Results

**Figure 1:**
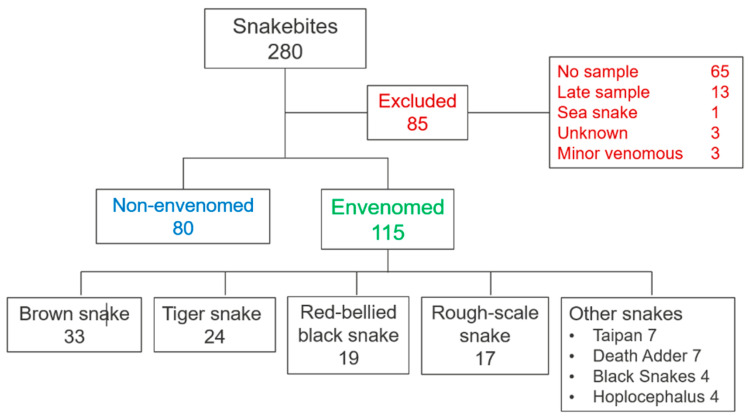
Flow chart showing the excluded patients (red) and the envenomated (green) and non-envenomated (blue) patients. Minor venomous snakes include two whip snake (*Demansia* spp.) bites and one bite by a De Vi’s Banded snake (*Denisonia devisi*).

We recruited 280 patients to the ASP over the two-year period with a median age of 37 years (interquartile range (IQR): 22 to 55 years; range: 2 to 82 years) and 190 (68%) were male. Eighty-five patients were excluded: 65 patients had no blood sample, 13 presented late, one was a sea snake envenomation, three patients were bitten by unknown snakes and three patients were bitten by minor venomous snakes (Figure 1). There were 115 envenomated patients and 80 non-envenomated patients. The most common snakes to cause envenomation were brown snakes (*Pseudonaja* spp., 33), then tiger snakes (*Notechis* spp., 24), red bellied black snakes (*Pseudechis porphyriacus*, 19) and rough-scaled snakes (*Tropidechis carinatus*, 17). The median time to the first blood sample in envenomated patients was 1.5 h (IQR: 1.0 to 2.4 h), compared to 1.7 (IQR: 1.0 to 3.0) for non-envenomated patients.

**Figure 2:**
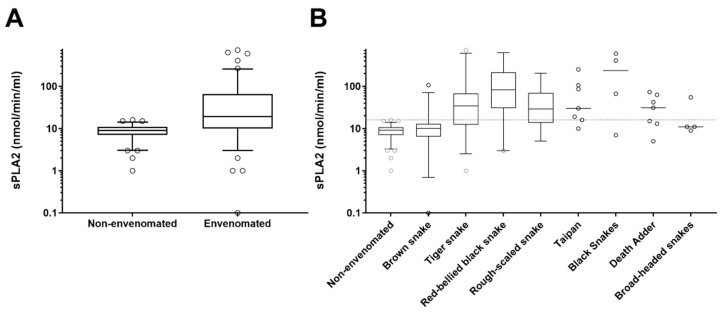
Box and whisker plots of the secretory phospholipase A_2_ concentrations for envenomated versus non-envenomated patients (**A**) and for non-envenomated patients and the different species of snakes (**B**). Scatter plots for the less common species. The boxes are medians and interquartile ranges. The gray dotted line represents the cut-off of 16 nmol/min/mL.

There was a significant difference in the median PLA_2_ activity between non-envenomated (9 nmol/min/mL; IQR: 7 to 11 nmol/min/mL) and envenomated patients (19 nmol/min/mL; IQR: 10 to 66 nmol/min/mL, *p* < 0.0001; Figure 2A). For the major groups of snake types, the median PLA_2_ activity for brown snakes was 10 nmol/min/mL (IQR: 6.5 to 113 nmol/min/mL), for tiger snakes, 34 nmol/min/mL (12 to 68 nmol/min/mL), for rough-scale snakes, 29 nmol/min/mL (14 to 69 nmol/min/mL) and red-bellied black snakes, 82 nmol/min/mL (30 to 212 nmol/min/mL), which were all significantly different to non-envenomated patients, except brown snake (Kruskal-Wallis *p* < 0.0001; Figure 2B and Table 1).

**Figure 3:**
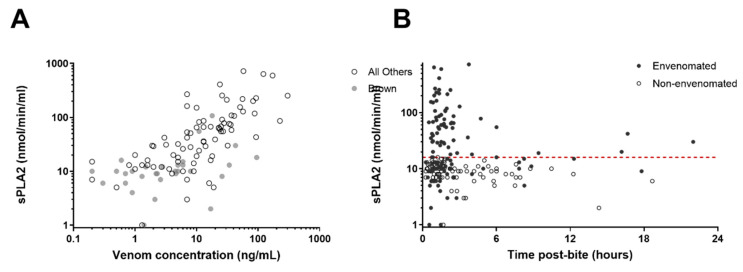
Plots of secretory phospholipase A_2_ concentrations versus venom concentration on double logarithmic axes (**A**) and secretory phospholipase A_2_ concentrations versus time on a logarithmic axis (**B**). The red dotted line represents the cut-off of 16 nmol/min/mL.

There was a significant correlation between venom concentration and PLA_2_ activity (r = 0.71; *p* < 0.0001) which was stronger when brown snake cases were excluded (Figure 3A). PLA_2_ activity was highest in the first 6 h post-bite for envenomated patients and then decreased over 24 h (Figure 3B).

**Figure 4:**
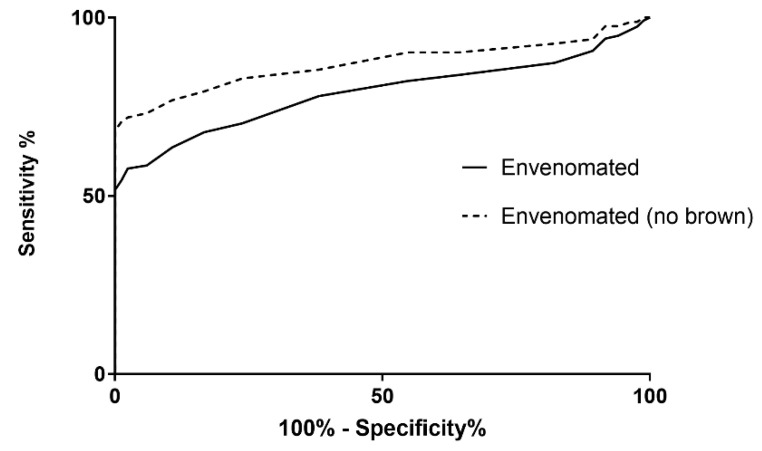
Area under the curve of the receiver operating curve for secretory phospholipase A_2_ concentrations for envenomated versus non-envenomated patients and non-envenomated patients versus envenomated patients (excluding brown snake).

PLA_2_ activity had a good predictive value for envenomation with an AUC-ROC of 0.79 (95% confidence intervals (95% CI): 0.72 to 0.85) but was excellent when brown snakes were excluded, AUC-ROC of 0.88 (95% CI: 0.82 to 0.94; Figure 4). A PLA_2_ activity of 16 nmol/min/mL was the optimal cut-point based on Youden’s index and had a 56% sensitivity (95% CI: 45 to 65%) and 99% specificity (95% CI: 93 to 100%) for identifying patients with systemic envenomation. Excluding patients with brown snakebites, a cut-point of 16 nmol/min/mL would be 72% sensitive (95% CI: 61 to 81%) and 99% specific (95% CI: 93 to 100%) for all other snakes, including all snakes that can potentially cause myotoxicity or neurotoxicity.

**Figure 5:**
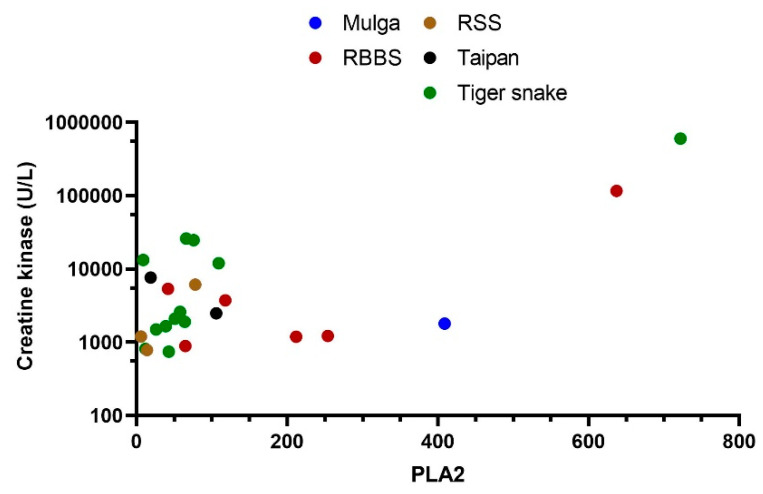
Plots of the peak creatine kinase (CK) versus secretory phospholipase A_2_ in patients with myotoxicity, including Mulga snake bites (*P. australis*), red-bellied black snake (*P. porphyriacus,* RBBS) bites, rough-scaled snake (*T. carinatus*; RSS) bites, taipan (*O. scutellatus*) bites and tiger snake (*Notechis* spp.) bites.

To further explore the relationship between PLA_2_ activity and toxicity, we compared the peak creatine kinase in patients with myotoxicity to PLA_2_ activity. There was a significant correlation between the peak CK and PLA_2_ (R2 = 0.46; *p* < 0.0001) in the five species of snakes that cause myotoxicity (*Notechis* spp., *P. australis*, *P. porphyriacus*, *T. carinatus* and *O. scutellatus*; Figure 5).

## 4. Discussion

We have shown that PLA_2_ activity is a good early predictor of systemic envenomation for Australian elapids, except brown snakes. An early PLA_2_ activity cut-off of 16 nmol/min/mL has an excellent specificity but poor sensitivity, so it would allow for early identification of envenomation but cannot exclude envenomation as a single test. Bloods were available for testing in about three-quarters of patients within 3 h of the bite, which would allow the administration of early antivenom if rapid testing were available. There was good correlation between PLA_2_ and both venom concentrations and the peak CK in patients with myotoxicity.

There are few previous studies investigating the association between PLA_2_ activity and snake envenomation [15]. Measurement of PLA_2_ activity was chosen as an early diagnostic test because it is an established assay and PLA_2_ are a major group of snake venom toxins present in most snakes [14]. Other potential important toxin groups are three-finger toxins, serine proteases and metalloproteases [14], but none of these have established assays for serum or plasma, or they are not enzymatic toxins.

We demonstrated that there was a strong association between PLA_2_ activity and venom concentration. This association was even stronger when brown snake envenomation cases were excluded, consistent with brown snake venom not containing much PLA_2_ activity. This demonstrates that the measurement of a single toxin enzyme activity was a valid method of detecting the presence of snake venom in serum. In addition, there was a significant correlation between PLA_2_ activity and peak creatine kinase, which is a surrogate measure of the severity of myotoxicity. Myotoxicity in Australian snake envenomation is due to PLA_2_ toxins in the venom [19]. This further supports the validity of this PLA_2_ activity assay as an indicator of venom being present in blood—systemic envenomation.

In this study, PLA_2_ activity was assayed at one central laboratory after samples were collected, frozen and stored. Currently, the assay must be undertaken in batches due to the available assay kits and it would not be possible for hospital laboratories to undertake this. In addition, the results of a PLA_2_ assay would need to be available within as short a period of time as possible (<60 min) for it to be useful in early antivenom decision making. Development of a simple PLA_2_ assay would be essential for the practical use of this test and appears to be possible based on some preliminary studies of point of care PLA_2_ assays using gold nanoparticles in a lateral flow assay or hybrid nanoparticles in a colorimetric assay [20,21].

Unfortunately, PLA_2_ activity measured in patients with brown snake envenomation was not significantly different to that in non-envenomated patients. This is consistent with the fact that brown snakes (*Pseudonaja* spp.) have low PLA_2_ activity compared to other Australian elapids [22]. In a practical sense, this means that PLA_2_ activity is not sensitive to brown snake envenomation and a low/normal value does not exclude brown snake envenomation. However, a PLA_2_ activity greater than 16 nmol/min/mL was highly specific for systemic envenomation and had a sensitivity of 72% for envenomation by snakes known to cause myotoxicity or neurotoxicity (*Notechis* spp. (tiger snakes), *Pseudechis* spp. (black snakes), *Tropidechis carinatus* (rough-scaled snake), *Oxyuranus scutellatus* (taipans) and *Ancanthophis* spp. (death adders); Figure 2).

Current Australian recommendations are that envenomated patients are treated with polyvalent antivenom (or two monovalent antivenoms to cover all possible snakes in a geographical region) [16,23]. A PLA_2_ activity greater than 16 nmol/min/mL in an early sample could potentially be used as an indication for antivenom. With such a high specificity, the risk of non-envenomed patients receiving antivenom would be negligible. Patients with a low PLA_2_ activity would still need to be observed and have further investigations.

A limitation of the study was the timing of the blood sample used for PLA_2_ testing. In almost all cases, the sample used was the admission blood sample and so the timing post-bite was dependent on the time it took the patient to arrive in hospital. Fortunately, the majority of blood samples were collected within 2.5 h (Figure 3A). This is likely to underestimate the diagnostic usefulness of the test because Figure 3A shows that the PLA_2_ is likely to be lower in later samples.

## 5. Conclusions

We have shown that the early measurement of PLA_2_ activity in Australian snakebites could be used to predict patients likely to develop complications of systemic envenomation and, therefore, guide the use of early antivenom. Unfortunately, the assay was not useful for brown snake envenomation and was not highly sensitive. Therefore, it has potential utility for early case identification (early rule in test) but may not be useful for excluding envenomation as a single test. The next step will be the development of rapid and point of care secretory PLA_2_ assays, which could be used at the bedside.

## Figures and Tables

**Table 1:** Phospholipase A_2_ activity and venom concentration of each of the snake types compared to non-envenomated patients.

Snake	No.	Phospholipase A_2_ (nmol/min/mL); Median, IQR and Range	Venom Concentration (ng/L); Median, IQR and Range
Non-envenomated	80	9 (7 to 11; 1 to 16)	NA
Brown snake (*Pseudonaja textilis*)	33	10 (6.5 to 13; 1 to 107)	2.6 (0.9 to 8; 0.2 to 95)
Tiger snake (*Notechis scutatus*)	24	34 (12 to 68; 1 to 68)	7 (2.2 to 25; 0.2 to 93)
Red-bellied black snake (*Pseudechis porphyriacus*)	19	82 (30 to 212; 3 to 637)	11 (3 to 51; 0.2 to 122)
Rough-scale snake (*Tropidechis carinatus*)	17	29 (14 to 69; 5 to 201)	14 (5.8 to 27; 0.5 to 83)
Taipan (*Oxyuranus scutellatus*)	7	30 (16 to 106; 10 to 252)	32 (17 to 227; 9 to 303)
Death Adder (*Acanthophis antarcticus*)	7	31 (13 to 63; 5 to 73)	7 (3.2 to 23; 1.3 to 36)
Mulga snake (*Pseudechis australis*)	3	7, 67, 409	7, 17, 24
Collett’s Snake (*Pseudechis colletti*)	1	597	173
Stephen’s banded snake (*Hoplocephalus stephensii*)	2	9, 55	25
Broad-headed snake (*Hoplocepalus bungaroides*)	2	11, 11	3.6

## References

[1] Longbottom J., Shearer F.M., Devine M., Alcoba G., Chappuis F., Weiss D.J., Ray S.E., Ray N., Warrell D.A., de Castañeda R.R. (2018). Vulnerability to snakebite envenoming: A global mapping of hotspots. Lancet.

[2] Kasturiratne A., Wickremasinghe A.R., de Silva N., Gunawardena N.K., Pathmeswaran A., Premaratna R., Savioli L., Lalloo D.G., de Silva H.J. (2008). The global burden of snakebite: A literature analysis and modelling based on regional estimates of envenoming and deaths. PLoS Med..

[3] Isbister G.K. (2010). Antivenom efficacy or effectiveness: The Australian experience. Toxicology.

[4] Johnston C.I., Ryan N.M., O’Leary M.A., Brown S.G., Isbister G.K. (2017). Australian taipan (*Oxyuranus* spp.) envenoming: Clinical effects and potential benefits of early antivenom therapy—Australian Snakebite Project (ASP-25). Clin. Toxicol..

[5] Churchman A., O’Leary M.A., Buckley N.A., Page C.B., Tankel A., Gavaghan C., Holdgate A., Brown S.G., Isbister G.K. (2010). Clinical effects of red-bellied black snake (*Pseudechis porphyriacus*) envenoming and correlation with venom concentrations: Australian Snakebite Project (ASP-11). Med. J. Aust..

[6] Lalloo D.G., Trevett A.J., Korinhona A., Nwokolo N., Laurenson I.F., Paul M., Black J., Naraqi S., Mavo B., Saweri A. (1995). Snake bites by the Papuan taipan (*Oxyuranus scutellatus* canni): Paralysis, hemostatic and electrocardiographic abnormalities, and effects of antivenom. Am. J. Trop. Med. Hyg..

[7] Silva A., Maduwage K., Sedgwick M., Pilapitiya S., Weerawansa P., Dahanayaka N.J., Buckley N.A., Johnston C., Siribaddana S., Isbister G.K. (2016). Neuromuscular Effects of Common Krait (*Bungarus caeruleus*) Envenoming in Sri Lanka. PLoS Negl. Trop. Dis..

[8] Johnston C.I., Brown S.G., O’Leary M.A., Currie B.J., Greenberg R., Taylor M., Barnes C., White J., Isbister G.K., ASP investigators (2013). Mulga snake (*Pseudechis australis*) envenoming: A spectrum of myotoxicity, anticoagulant coagulopathy, haemolysis and the role of early antivenom therapy—Australian Snakebite Project (ASP-19). Clin. Toxicol..

[9] Kularatne S.A., Silva A., Weerakoon K., Maduwage K., Walathara C., Paranagama R., Mendis S. (2014). Revisiting Russell’s viper (Daboia russelii) bite in Sri Lanka: Is abdominal pain an early feature of systemic envenoming?. PLoS ONE.

[10] Isbister G.K., Scorgie F.E., O’leary M.A., Seldon M., Brown S.G., Lincz L.F., ASP Investigators (2010). Factor deficiencies in venom-induced consumption coagulopathy resulting from Australian elapid envenomation: Australian Snakebite Project (ASP-10). J. Thromb. Haemost..

[11] Isbister G.K., Maduwage K., Shahmy S., Mohamed F., Abeysinghe C., Karunathilake H., Ariaratnam C.A., Buckley N.A. (2013). Diagnostic 20-min whole blood clotting test in Russell’s viper envenoming delays antivenom administration. QJM.

[12] Ratnayake I., Shihana F., Dissanayake D.M., Buckley N.A., Maduwage K., Isbister G.K. (2017). Performance of the 20-minute whole blood clotting test in detecting venom induced consumption coagulopathy from Russell’s viper (Daboia russelii) bites. Thromb. Haemost..

[13] Johnston C., Isbister G.K. (2020). Australian Snakebite Myotoxicity (ASP-23). Clin. Toxicol..

[14] Tasoulis T., Isbister G.K. (2017). A Review and Database of Snake Venom Proteomes. Toxins.

[15] Maduwage K., O’Leary M.A., Isbister G.K. (2014). Diagnosis of snake envenomation using a simple phospholipase A2 assay. Sci. Rep..

[16] Johnston C.I., Ryan N.M., Page C.B., Buckley N.A., Brown S.G., O’Leary M.A., Isbister G.K. (2017). The Australian Snakebite Project, 2005–2015 (ASP-20). Med. J. Aust..

[17] Ireland G., Brown S.G., Buckley N.A., Stormer J., Currie B.J., White J., Spain D., Isbister G.K. (2010). Changes in serial laboratory test results in snakebite patients: When can we safely exclude envenoming?. Med. J. Aust..

[18] Kulawickrama S., O’Leary M.A., Hodgson W.C., Brown S.G., Jacoby T., Davern K., Isbister G.K. (2010). Development of a sensitive enzyme immunoassay for measuring taipan venom in serum. Toxicon.

[19] Hart A.J., Hodgson W.C., O’Leary M., Isbister G.K. (2014). Pharmacokinetics and pharmacodynamics of the myotoxic venom of *Pseudechis australis* (mulga snake) in the anesthetised rat. Clin. Toxicol..

[20] Chapman R., Lin Y., Burnapp M., Bentham A., Hillier D., Zabron A., Khan S., Tyreman M., Stevens M.M. (2015). Multivalent nanoparticle networks enable point-of-care detection of human phospholipase-A2 in serum. ACS Nano.

[21] Aili D., Mager M., Roche D., Stevens M.M. (2011). Hybrid nanoparticle-liposome detection of phospholipase activity. Nano Lett..

[22] Tasoulis T., Lee M.S.Y., Ziajko M., Dunstan N., Sumner J., Isbister G.K. (2020). Activity of two key toxin groups in Australian elapid venoms show a strong correlation to phylogeny but not to diet. BMC Evol. Biol..

[23] Isbister G.K., Brown S.G., Page C.B., McCoubrie D.L., Greene S.L., Buckley N.A. (2013). Snakebite in Australia: A practical approach to diagnosis and treatment. Med. J. Aust..

